# Adverse outcomes after non-hepatic surgeries in patients with alcoholic liver diseases: a propensity-score matched study

**DOI:** 10.1186/s12876-022-02558-6

**Published:** 2022-11-21

**Authors:** Hsin-Yun Wu, Chuen-Chau Chang, Chun-Chieh Yeh, Ming-Yao Chen, Yih-Giun Cherng, Ta-Liang Chen, Chien-Chang Liao

**Affiliations:** 1 Department of Anesthesiology, Shuang Ho Hospital, Taipei Medical University, New Taipei City, Taiwan; 2 Department of Anesthesiology, School of Medicine, College of Medicine, Taipei Medical University, Taipei, Taiwan; 3 Anesthesiology and Health Policy Research Center, Taipei Medical University Hospital, Taipei, Taiwan; 4 Department of Anesthesiology, Taipei Medical University Hospital, 252 Wuxing St., Taipei, 11031 Taiwan; 5 Team of Liver Transplantation, Department of Surgery, China Medical University Hospital, Taichung, Taiwan; 6 Department of Surgery, University of Illinois, Chicago, USA; 7 Division of Gastroenterology and Hepatology, Department of Internal Medicine, Shuang Ho Hospital, Taipei Medical University, New Taipei City, Taiwan; 8 Department of Anesthesiology, Wan Fang Hospital, Taipei Medical University, Taipei, Taiwan; 9 Research Center of Big Data and Meta-Analysis, Wan Fang Hospital, Taipei Medical University, Taipei, Taiwan; 10 Centers of Regional Anesthesia and Pain Medicine, Wan Fang Hospital, Taipei Medical University, Taipei, Taiwan; 11 School of Chinese Medicine, College of Chinese Medicine, China Medical University, Taichung, Taiwan

**Keywords:** Alcoholic liver diseases, Non-hepatic surgeries, Adverse outcomes

## Abstract

**Background:**

The influence of alcoholic liver disease (ALD) on the postoperative outcomes is not completely understood. Our purpose is to evaluate the complications and mortality after nonhepatic surgeries in patients with ALD.

**Methods:**

We conducted a retrospective cohort study included adults aged 20 years and older who underwent nonhepatic elective surgeries using data of Taiwan’s National Health Insurance, 2008–2013. Using a propensity-score matching procedure, we selected surgical patients with ALD (n = 26,802); or surgical patients without ALD (n = 26,802) for comparison. Logistic regression was used to calculate the odds ratios (ORs) and 95% confidence intervals (CIs) of postoperative complications and in-hospital mortality associated with ALD.

**Results:**

Patients with ALD had higher risks of acute renal failure (OR 2.74, 95% CI 2.28–3.28), postoperative bleeding (OR 1.64, 95% CI 1.34–2.01), stroke (OR 1.51, 95% CI 1.34–1.70) septicemia (OR 1.47, 95% CI 1.36–1.58), pneumonia (OR 1.43, 95% CI 1.29–1.58), and in-hospital mortality (OR 2.64, 95% CI 2.24–3.11) than non-ALD patients. Patients with ALD also had longer hospital stays and higher medical expenditures after nonhepatic surgical procedures than the non-ALD patients. Compared with patients without ALD, patients with ALD who had jaundice (OR 4.82, 95% CI 3.68–6.32), ascites (OR 4.57, 95% CI 3.64–5.74), hepatic coma (OR 4.41, 95% CI 3.44–5.67), gastrointestinal hemorrhage (OR 3.84, 95% CI 3.09–4.79), and alcohol dependence syndrome (OR 3.07, 95% CI 2.39–3.94) were more likely to have increased postoperative mortality.

**Conclusion:**

Surgical patients with ALD had more adverse events and a risk of in-hospital mortality after nonhepatic surgeries that was approximately 2.6-fold higher than that for non-ALD patients. These findings suggest the urgent need to revise the protocols for peri-operative care for this population.

**Supplementary Information:**

The online version contains supplementary material available at 10.1186/s12876-022-02558-6.

## Introduction

Alcohol is commonly consumed in surgical patients during the perioperative period [1, 2]. The prevalence of alcohol consumption during the year before surgery is approximately 5–16%, with the level of alcohol consumption recognized as severe misuse in 2–4% of these patients [1], even though there are many unrecorded users [2]. Previous studies suggested that alcohol consumption during the perioperative period may increase complications and medical expenditures after surgery [3, 4].

Previous reports indicated that acute alcohol use was more harmful than chronic alcohol misuse among patients who underwent surgery [5]. In contrast, reducing the consumption of alcohol during the perioperative period improves postoperative outcomes [6]. A systematic review and meta-analysis including 55 studies suggested that preoperative alcohol consumption was associated with risks of postoperative morbidities, general infections, wound complications, pulmonary complications, prolonged hospital stays, and intensive care unit admission [7]. However, another meta-analysis suggested that alcohol drinking was not a risk factor for surgical site infection and anastomotic leakage [8].

These inconclusive findings imply the need for further research. In addition, the effects of alcoholic liver disease (ALD) on perioperative outcomes are not completely understood [1, 3, 7, 8]. Therefore, we used claims data from Taiwan’s National Health Insurance to explore the outcomes after nonhepatic surgeries in patients with and without alcoholic liver disease (ALD).

## Methods

### Source of data

We used the reimbursement claims data from the Taiwan’s National Health Insurance Program, which was implemented in March 1995 and covers more than 99% of the 23 million Taiwan residents [9–12]. The National Health Research Institutes of Taiwan established a National Health Insurance Research Database that records all beneficiaries’ medical services, including inpatient and outpatient demographics, primary and secondary diagnoses, procedures, prescriptions and medical expenditures for public research interest [9–12]. The validation of Taiwan’s National Health Insurance Research Database has been evaluated in previous studies [9, 10]. The validity of this database has been favorably evaluated, and research articles based on it have been accepted in prominent scientific journals worldwide [11, 12].

The data that support the findings of this study are available from the Ministry of Health and Welfare, Taiwan but restrictions apply to the availability of these data, which were used under license for the current study, and so are not publicly available. Data are however available from the authors upon reasonable request and with permission of the Ministry of Health and Welfare, Taiwan. We have made the formal application (included application documents, study proposals, and ethics approval of the institutional review board) of the current insurance data. The authors of the present study had no special access privileges in accessing the data which other interested researchers would not have [11, 12].

We conducted this study in accordance with the Helsinki Declaration. In the original insurance data, every patient had identification number. For protecting personal privacy, the Ministry of Health and Welfare decoded the identification number in the insurance research database. Therefore, the researchers used insurance research database could not understand the identification of patients and they also could not identify specific patients. The requirement of informed consent to participate was deemed unnecessary according to the regulations of the Ministry of Health and Welfare. The requirement of informed consent to participate was waived by the Institutional Review Board of Taipei Medical University that also evaluated and approved this study (TMU-JIRB-202203134; TMU-JIRB-201905042; TMU-JIRB-201902053; TMU-JIRB-201705063).

### Study design

From the three million surgical patients who underwent nonhepatic elective surgeries between 2008 and 2013 in Taiwan, we identified 32,548 surgical patients with ALD who were aged 20 years and older. These elective surgeries were nonhepatic surgeries that required general, epidural, or spinal anesthesia and hospitalization for at least 1 day. To identify patients with ALD more clearly, the current study required at least one medical care visit with a physician’s diagnosis of ALD within the 24-month preoperative period of the index surgery. Using a matching propensity score procedure with age, sex, low income, hospital volume, types of surgery, types of anesthesia, number of inpatient care visits within the past 2 years, number of emergency care visits within the past 2 years, and coexisting medical conditions (including mental disorders, hypertension, diabetes, peptic ulcer disease, chronic obstructive pulmonary disease, ischemic heart disease, hyperlipidemia, chronic kidney disease, heart failure, and renal dialysis), we selected 32,548 surgical patients from the surgical patient populations who were without ALD preoperatively.

### Measures and definitions

We identified income status by defining low-income patients as those qualifying for waived medical copayment because this status is verified by the Taiwan Bureau of National Health Insurance. Additionally, whether the surgery was performed in a teaching hospital and the types of surgery and anesthesia used were also recorded. In this study, we excluded sucgrical patients received hepatic surgeries, such as wedge biopsy of liver, partial hepatectomy, segemental hepatectomy, hepatorrhaphy, hepato-enterostomy, portocavo shunt, Warren's shunt, right lobectomy, left lobectomy, and liver transplantation. The details of procedure codes were showed in Additional file 1: Table S1.

We used the International Classification of Diseases, Ninth Revision, Clinical Modification (ICD-9-CM) to define preoperative medical diseases and postoperative complications. Preoperative ALD was defined as the main exposure and included alcoholic fatty liver (ICD-9-CM 571.0), acute alcoholic hepatitis (ICD-9-CM 571.1), alcoholic cirrhosis of the liver (ICD-9-CM 571.2), and alcoholic liver damage (ICD-9-CM 571.3). Coexisting medical conditions, including mental disorders (ICD-9-CM 290-319), hypertension (ICD-9-CM401-405), diabetes (ICD-9-CM 250), peptic ulcer disease (ICD-9-CM 531, 532, 533), chronic obstructive pulmonary disease (ICD-9-CM 491, 492, 496), ischemic heart disease (ICD-9-CM 401-414), hyperlipidemia (ICD-9-CM 272.0, 272.1, 272.2), chronic kidney disease (ICD-9-CM 585, 586), heart failure (ICD-9-CM 428), and renal dialysis (administration codes D8, D9), were determined from medical claims for the 24-month preoperative period. Jaundice (ICD-9-CM 782.4), hepatic coma (ICD-9-CM 572.2), gastrointestinal hemorrhage (ICD-9-CM 578), ascites (ICD-9-CM 789.5) and alcohol dependence syndrome (ICD-9-CM 303) were also identified as clinical symptoms of patients with ALD.

Thirty-day in-hospital mortality after the index surgery and postoperative complications were considered as the study’s outcomes. These complications included septicemia (ICD-9-CM 038 and 998.5), pneumonia (ICD-9-CM 480-486), urinary tract infection (ICD-9-CM 599.0), acute renal failure (ICD-9-CM 584), stroke (ICD-9-CM 430-438), deep wound infection (ICD-9-CM 958.3), postoperative bleeding (ICD-9-CM 998.0, 998.1 and 998.2) and pulmonary embolism (ICD-9-CM 415). Admission to the intensive care unit, length of hospital stay and medical expenditure during the index nonhepatic surgery were also considered as secondary outcomes in this study.

### Statistical analysis

We used a nonparsimonious multivariable logistic regression model to estimate propensity scores for preoperative ALD, irrespective of outcome. Clinical significance guided the initial choice of covariates in this model: age, sex, low income, hospital volume, types of surgery, types of anesthesia, number of inpatient care visits within the past 2 years, number of emergency care visits within the past 2 years, mental disorders, hypertension, diabetes, peptic ulcer disease, chronic obstructive pulmonary disease, ischemic heart disease, hyperlipidemia, chronic kidney disease, heart failure, and renal dialysis. We used a structured iterative approach to refine this model with the goal of achieving covariate balance within the matched pairs. Chi-square tests were used to measure covariate balance, and *p* < 1.0 was suggested to represent covariate imbalance. We matched patients with ALD to patients without ALD using a greedy-matching algorithm with a caliper width of 0.2 standard deviation of the log odds of the estimated propensity score. This method has been estimated to remove 98% of the bias from measured covariates. Adjusted odds ratios (ORs) with 95% CIs for 30-day postoperative complications and mortality between patients with and without ALD were analyzed with multivariate logistic regression. We controlled for age, sex, low income, hospital volume, types of surgery, types of anesthesia, number of inpatient care visits within the past 2 years, number of emergency care visits within the past 2 years, mental disorders, hypertension, diabetes, peptic ulcer disease, chronic obstructive pulmonary disease, ischemic heart disease, hyperlipidemia, chronic kidney disease, heart failure, and renal dialysis. We performed a stratified analysis and calculated an adjusted HR and 95% CI to examine the association between ALD and 30-day in-hospital mortality after surgery in the multivariate logistic regressions. SAS version 9.1 (SAS Institute Inc., Cary, NC, USA) statistical software was used; two-sided *p* < 0.05 indicated significant differences between surgical patients with and without ALD.

## Results

Table 1 shows the matched characteristics of surgical patients with and without ALD. There was no difference in age, sex, low income, hospital volume, types of surgery, types of anesthesia, inpatient care visits within the past 2 years, emergency care visits within the past 2 years, mental disorders, hypertension, diabetes, peptic ulcer disease, chronic obstructive pulmonary disease, ischemic heart disease, hyperlipidemia, chronic kidney disease, heart failure, or renal dialysis between the ALD patients and non-ALD patients. The liver-related characteristics of surgical patients with and without preoperative ALD was showed in Additional file 1: Table S2.

**Table 1:** Characteristics of surgical patients with and without preoperative alcoholic liver disease

	Alcoholic liver disease				*p* value
	No (N = 26,802)		Yes (N = 26,802)		
Age, years	n	(%)	n	(%)	1.0000
20–29	670	(2.5)	670	(2.5)	
30–39	4184	(15.6)	4184	(15.6)	
40–49	9047	(33.8)	9047	(33.8)	
50–59	8294	(31.0)	8294	(31.0)	
60–69	3315	(12.4)	3315	(12.4)	
≥ 70	1292	(4.8)	1292	(4.8)	
Mean ± SD	50.1 ± 11.8		49.9 ± 11.1		0.1285
Median (IQR)	49.5 (15.0)		49.5 (14.5)		0.2749
Sex					1.0000
Male	24,250	(90.5)	24,250	(90.5)	
Female	2552	(9.5)	2552	(9.5)	
Low income					1.0000
No	26,146	(97.6)	26,146	(97.6)	
Yes	656	(2.4)	656	(2.4)	
Volume of hospital					1.0000
Low	7302	(27.2)	7302	(27.2)	
Medium	8906	(33.2)	8906	(33.2)	
High	10,594	(39.5)	10,594	(39.5)	
Medical conditions					
Mental disorders	5151	(19.2)	5151	(19.2)	1.0000
Hypertension	3981	(14.9)	3981	(14.9)	1.0000
Diabetes	2779	(10.4)	2779	(10.4)	1.0000
Peptic ulcer disease	2172	(8.1)	2172	(8.1)	1.0000
COPD	593	(2.2)	593	(2.2)	1.0000
Ischemic heart disease	618	(2.3)	618	(2.3)	1.0000
Hyperlipidemia	719	(2.7)	719	(2.7)	1.0000
Chronic kidney disease	67	(0.3)	67	(0.3)	1.0000
Heart failure	55	(0.2)	55	(0.2)	1.0000
Renal dialysis	36	(0.1)	36	(0.1)	1.0000
Inpatient care in past 2 years					1.0000
0	13,872	(51.8)	13,872	(51.8)	
1	7356	(27.5)	7356	(27.5)	
2	2486	(9.3)	2486	(9.3)	
≥ 3	3088	(11.5)	3088	(11.5)	
Emergency care in past 2 years					1.0000
0	13,134	(49.0)	13,134	(49.0)	
1	6799	(25.4)	6799	(25.4)	
2	3063	(11.4)	3063	(11.4)	
≥ 3	3806	(14.2)	3806	(14.2)	
Types of surgery					1.0000
Digestive	8914	(33.3)	8914	(33.3)	
Musculoskeletal	8978	(33.5)	8978	(33.5)	
Neurosurgery	2850	(10.6)	2850	(10.6)	
Kidney, Ureter, Bladder	1922	(7.2)	1922	(7.2)	
Respiratory	1543	(5.8)	1543	(5.8)	
Cardiovascular	391	(1.5)	391	(1.5)	
Skin	601	(2.2)	601	(2.2)	
Breast	79	(0.3)	79	(0.3)	
Delivery, CS, Abortion	229	(0.9)	229	(0.9)	
Eye	124	(0.5)	124	(0.5)	
Others	1171	(4.4)	1171	(4.4)	
Types of anesthesia					1.0000
General	21,119	(78.8)	21,119	(78.8)	
Epidural or Spinal	5683	(21.2)	5683	(21.2)	

After adjusting for all covariates listed in Table 1 using multivariate logistic regression analyses (Table 2), we found that patients with ALD had higher risks of postoperative acute renal failure (OR 2.74, 95% CI 2.28–3.28), postoperative bleeding (OR 1.64, 95% CI 1.34–2.01), stroke (OR 1.51, 95% CI 1.34–1.70), septicemia (OR 1.47, 95% CI 1.36–1.58), pneumonia (OR 1.43, 95% CI 1.29–1.58), and 30-day in-hospital mortality (OR 2.64, 95% CI 2.24–3.11) compared with patients without ALD. The medical expenditures (3548 ± 6578 vs. 2939 ± 5187 US dollars; *p* < 0.0001) and hospital length of stay (10.9 ± 15.1 vs. 10.0 ± 15.8 days; *p* < 0.0001) of the index surgical admission were comparatively greater for the ALD patients than the non-ALD patients.

**Table 2:** Risks of complications and mortality after non-hepatic surgeries for patients with and without alcoholic liver disease

	No ALD (N = 26,802)		ALD (N = 26,802)		Risk of outcomes	
	Events	%	Event	%	OR	(95% CI)^a^
30-day in-hospital mortality	204	0.8	523	2.0	2.64	(2.24–3.11)
Postoperative complications						
Acute renal failure	165	0.6	438	1.6	2.74	(2.28–3.28)
Postoperative bleeding	155	0.6	253	0.9	1.64	(1.34–2.01)
Stroke	505	1.9	725	2.7	1.51	(1.34–1.70)
Septicemia	1287	4.8	1825	6.8	1.47	(1.36–1.58)
Pneumonia	701	2.6	974	3.6	1.43	(1.29–1.58)
Deep wound infection	208	0.8	241	0.9	1.16	(0.96–1.40)
Pulmonary embolism	14	0.1	17	0.1	1.22	(0.60–2.47)
Urinary tract infection	720	2.7	741	2.8	1.03	(0.93–1.15)
Length of stay, days^b^	2939 ± 5187		3548 ± 6578		*p* < 0.0001	
Medical expenditure, US dollars^b^	10.0 ± 15.8		10.9 ± 15.1		*p* < 0.0001	

ALD, Alcoholic liver disease; CI, confidence interval; OR, odds ratio

^a^Adjusted for all covariates listed in Table 1

^b^Mean ± SD

In Table 3, the association between ALD and 30-day in-hospital mortality after nonhepatic surgeries was more significant in females (OR 4.07, 95% CI 1.63–10.1) than in males (OR 2.60, 95% CI 2.20–3.07). The 30-day in-hospital mortality after nonhepatic surgeries was associated with ALD in patients aged 30–39 years (OR 3.26, 95% CI 1.88–5.66), 40–49 years (OR 3.04, 95% CI 2.34–3.96), 50–59 years (OR 2.76, 95% CI 2.05–3.70), and 60–69 years (OR 2.00, 95% CI 1.26–3.17). The adjusted ORs for the association between ALD and 30-day in-hospital mortality after nonhepatic surgeries for patients with 0, 1, 2, and ≥ 3 medical conditions were 2.79 (95% CI 2.24–3.49), 2.70 (95% CI 2.06–3.55), 1.49 (95% CI 0.83–2.70), and 2.65 (95% CI 0.49–14.3), respectively.

**Table 3:** The stratification analysis for the association between alcoholic liver disease 30-day in-hospital mortality

	n	30-day in-hospital mortality			
		Deaths	Mortality, %	OR	(95% CI)^a^
Female					
No ALD	2552	6	0.2	1.00	(reference)
ALD	2552	23	0.9	4.07	(1.63–10.1)
Male					
No ALD	24,250	198	0.8	1.00	(reference)
ALD	24,250	500	2.1	2.60	(2.20–3.07)
Age 20–29 years					
No ALD	670	0	0.0	1.00	(reference)
ALD	670	4	0.6	–	–
Age 30–39 years					
No ALD	4184	17	0.4	1.00	(reference)
ALD	4184	54	1.3	3.26	(1.88–5.66)
Age 40–49 years					
No ALD	9047	77	0.9	1.00	(reference)
ALD	9047	224	2.5	3.04	(2.34–3.96)
Age 50–59 years					
No ALD	8294	62	0.8	1.00	(reference)
ALD	8294	166	2.0	2.76	(2.05–3.70)
Age 60–69 years					
No ALD	3315	28	0.8	1.00	(reference)
ALD	3315	55	1.7	2.00	(1.26–3.17)
Age ≥ 70 years					
No ALD	1292	20	1.6	1.00	(reference)
ALD	1292	20	1.6	1.00	(0.53–1.89)
0 medical condition					
No ALD	13,871	110	0.8	1.00	(reference)
ALD	13,871	298	2.2	2.79	(2.24–3.49)
1 medical condition					
No ALD	10,067	73	0.7	1.00	(reference)
ALD	10,067	192	1.9	2.70	(2.06–3.55)
2 medical conditions					
No ALD	2521	19	0.8	1.00	(reference)
ALD	2521	28	1.1	1.49	(0.83–2.70)
≥ 3 medical conditions					
No ALD	343	2	0.6	1.00	(reference)
ALD	343	5	1.5	2.65	(0.49–14.3)
Digestive surgery					
No ALD	8914	93	1.0	1.00	(reference)
ALD	8914	208	2.3	2.28	(1.78–2.92)
Musculoskeletal surgery					
No ALD	8978	23	0.3	1.00	(reference)
ALD	8978	73	0.8	3.21	(2.01–5.14)
Neurosurgery surgery					
No ALD	2850	51	1.8	1.00	(reference)
ALD	2850	164	5.8	3.38	(2.46–4.66)
Kidney, Ureter, Bladder surgery					
No ALD	1922	10	0.5	1.00	(reference)
ALD	1922	13	0.7	1.31	(0.57–3.01)
Respiratory surgery					
No ALD	1543	7	0.5	1.00	(reference)
ALD	1543	13	0.8	1.89	(0.74–4.78)
Cardiovascular surgery					
No ALD	391	14	3.6	1.00	(reference)
ALD	391	36	9.2	2.89	(1.51–5.53)
Skin surgery					
No ALD	601	1	0.2	1.00	(reference)
ALD	601	5	0.8	5.31	(0.60–46.8)
Breast surgery					
No ALD	79	0	0.0	1.00	(reference)
ALD	79	1	1.3	–	–
Delivery, CS, Abortion surgery					
No ALD	229	0	0.0	1.00	(reference)
ALD	229	1	0.4	–	–
Eye surgery					
No ALD	124	1	0.8	1.00	(reference)
ALD	124	1	0.8	1.00	(0.04–24.4)
Others surgery					
No ALD	1171	4	0.3	1.00	(reference)
ALD	1171	8	0.7	2.09	(0.61–7.22)

CI, confidence interval; OR, odds ratio

^a^Adjusted for all covariates listed in Table 1

Further analysis (Table 4) of the correlation between 30-day in-hospital mortality after nonhepatic surgeries and different severities of ALD showed that acute alcoholic hepatitis (OR 1.96, 95% CI 1.47–2.62), alcoholic cirrhosis of the liver (OR 3.94, 95% CI 3.30–4.70), alcoholic liver damage (OR 2.03, 95% CI 1.64–2.52), and suffering more than two alcoholic liver diseases (OR 2.64, 95% CI 2.01–3.48) were associated with 30-day in-hospital mortality. For patients with ALD, alcohol dependence syndrome (OR 3.07, 95% CI 2.39–3.94), jaundice (OR 4.82, 95% CI 3.68–6.32), ascites (OR 4.57, 95% CI 3.64–5.74), gastrointestinal hemorrhage (OR 3.84, 95% CI 3.09–4.79), hepatic coma (OR 4.41, 95% CI 3.44–5.67), and ≥ 2 types of alcoholic liver diseases (OR 4.58, 95% CI 3.67–5.71) were significant factors contributing to 30-day in-hospital mortality after nonhepatic surgeries.

**Table 4:** Risks of postoperative mortality for surgical patients with the severity of alcoholic liver disease

	n	30-day in-hospital mortality			
		Deaths	Mortality, %	OR	(95% CI)^a^
Non-ALD controls	26,802	204	0.8	1.00	(reference)
Patients with					
Alcoholic fatty liver	5557	31	0.6	1.03	(0.70–1.51)
Acute alcoholic hepatitis	4438	62	1.4	1.96	(1.47–2.62)
Alcoholic liver damage, unspecified	9600	144	1.5	2.03	(1.64–2.52)
Alcoholic cirrhosis of liver	10,579	366	3.5	3.94	(3.30–4.70)
≥ 2 alcoholic liver diseases	3176	73	2.3	2.64	(2.01–3.48)
ALD patients with					
Alcohol dependence syndrome	3880	106	2.7	3.07	(2.39–3.94)
Gastrointestinal hemorrhage	4226	155	3.7	3.84	(3.09–4.79)
Hepatic coma	2265	109	4.8	4.41	(3.44–5.67)
Ascites	3040	143	4.7	4.57	(3.64–5.74)
Jaundice	1743	81	4.7	4.82	(3.68–6.32)
≥ 2 the above indicators	3619	167	4.6	4.58	(3.67–5.71)
Chronic hepatitis	12,509	249	2.0	2.70	(2.24–3.26)
Liver cirrhosis	12,409	367	3.0	3.58	(3.00–4.26)
Liver cancer	1504	34	2.3	2.08	(1.43–3.04)
Patients with ALD diagnosis within					
Preoperative 1–3 month	4091	85	2.1	2.63	(2.03–3.41)
Preoperative 4–6 month	2551	53	2.1	2.60	(1.91–3.54)
Preoperative 7–12 month	4705	87	1.9	2.42	(1.87–3.13)

CI, confidence interval; OR, odds ratio

^a^Adjusted for all covariates listed in Table 1

Additional file 1: Table S3 showed the risks of postoperative mortality for surgical patients with the severity of alcoholic liver disease. Additional file 1: Table S4 showed the risks of postoperative adverse events for surgical patients with the severity of alcoholic liver disease.

## Discussion

This is the first nationwide population-based study to discuss the postoperative risks in patients with ALD receiving nonhepatic surgery and present the association between ALD and 30-day in-hospital mortality. Patients with ALD displayed higher risks of postoperative acute renal failure, postoperative bleeding, postoperative stroke, postoperative septicemia, postoperative pneumonia, 30-day in-hospital mortality, length of stay, and medical expenditures compared with patients without ALD who underwent nonhepatic surgeries.

There were several strengths in this study, such as large sample size, propensity-score matching, validated database, and integrative assessment for postoperative outcomes. However, the study limitations suggest that caution should be taken when interpreting our study findings. First, physical examinations, laboratory data, and the patients’ sociodemographic and lifestyle characteristics could not be extracted from the reimbursement data in Taiwan’s National Health Insurance Research Database. Thus, we could not evaluate the influence of these factors on the perioperative outcome in patients with ALD. Second, we have no information regarding the severity of ALD, such as the Child–Pugh score or the Model score for End-stage Liver Disease score [13, 14]. Third, to identify ALD cases correctly, we required at least 2 medical care visits with a physician’s primary diagnosis of ALD for inclusion. We could not exclude the possibility that some ALD cases without medical care may have been included in the control group. Fourth, there were no details on the alcohol drinking status of participants because of the unavailable information in the claim database. We admitted this is also one of study limitations. Fifth, although the procedure and diagnosis codes of ALD has not been validated in the previous studies, we considered that the codes used in this study is reliable because the insurance claims and payment was strictly examined and reviewed by the Ministry of Health and Welfare, Taiwan. In addition, we could not analyze the duration of ALD for more the two years because of the unavailable data of insurance database. We also could not evaluate the influence of emergency surgeries on the postoperative outcomes in patients with ALD because we have no related information of emergency surgeries in this study. Finally, residual confounding bias may be possible, although we used propensity-score matching and multivariate regression adjustments.

To our knowledge, advanced age, sex, low income, hospital volume, medical conditions (such as mental disorders, hypertension, diabetes, peptic ulcer disease, chronic obstructive pulmonary disease, ischemic heart disease, hyperlipidemia, chronic kidney disease, heart failure and renal failure), type of surgery, and type of anesthesia were associated with postoperative outcomes [15–18]. After adjusting for these confounding factors, our findings suggest that ALD increased the risks of postoperative complications and 30-day in-hospital mortality following nonhepatic surgery.

Some possible reasons may help to explain the association between ALD and postoperative complications. First, acute renal failure is a common presentation in patients hospitalized for advanced liver cirrhosis with acute decompensation. Hepatorenal syndrome is one of the comorbidities of end-stage liver disease due to severe portal hypertension [19]. Moreover, during the perioperative period, prerenal hypoperfusion is worsened by the hypotension induced by anesthesia or surgical blood loss. Therefore, postoperative acute renal failure is more common in patients with alcoholic liver disease. Second, coagulation and thrombocytopenia are common problems in ALD patients [20]. Spontaneous deep bleeding into muscles and the retroperitoneum due to disordered and unstable coagulation has been reported [21]. Obviously, more postoperative bleeding problems are expected in patients with ALD than in patients without ALD. Third, some studies have reported that liver cirrhosis may cause endothelial dysfunction and chronic inflammation [22, 23]; as a result, both were suggested to be associated with increased ischemic and hemorrhagic stroke risks [11]. Fourth, chronic inflammation usually occurs in patients with ALD due to hepatic steatosis, oxidative stress, and acetaldehyde-mediated toxicity [24]. The long-term inflammation problem induced by cytokines and chemokines may impair the autoimmune system and may be associated with increased risks of septicemia and pneumonia [25]. Fifth, preoperative alcohol consumption is related to intraoperative and postoperative delirium, which will affect the sensitivity of symptom recognition and quality of care [26]. ALD patients usually have poor self-care abilities, family support, health knowledge, attitudes, and practices of disease prevention and treatments [26, 27]. Malnutrition is common in patients with chronic liver disease, which can also explain the poorer prognosis in ALD patients [28]. In addition, the comorbidities of patients with ALD, such as liver cirrhosis, encephalopathy, and ascites, may contribute to postoperative complications [12, 29]. Acute chronic liver failure presenting in advanced stages, precipitated by some special events, including bacterial infection, acute alcoholic, and drug-induced or viral hepatitis, is associated with increased morbidities and mortality [30]. These factors explain how ALD patients have worse postoperative complications, longer hospital length of stay, more medical expenditure, and increased mortality.

Our study suggested that ALD increases 30-day in-hospital mortality at all ages, especially in women. Sex-related differences in inflammatory and immune biomarker concentrations in ALD patients were reported [31]. Previous studies revealed that female drinkers are at greater risk of ALD than men due to their increased likelihood of developing acute liver failure from excessive alcohol use and more rapid progression of ALD [32]. Patients with ALD have an approximately 2.6-fold increased risk of 30-day in-hospital mortality, regardless of the number of medical conditions. This result implied that ALD affects patient mortality more than other diseases. In the subgroup analysis, ALD patients with acute alcoholic hepatitis, alcoholic liver damage, alcoholic liver cirrhosis and more than two alcoholic liver diseases had a 2–4-fold increase in 30-day in-hospital mortality. Among those different types of alcoholic liver disease, alcoholic liver cirrhosis most affected mortality in patients who underwent nonhepatic surgery. There are systemic comorbidities related to cirrhosis, such as hepatic encephalopathy, hepatorenal syndrome, and cirrhotic cardiomyopathy [19, 30, 33]. These comorbidities and their associated systemic organ damage may greatly affect surgical patient mortality following nonhepatic surgery. Furthermore, the ORs for 30-day in-hospital mortality were higher in ALD patients with alcohol dependence syndrome, gastrointestinal hemorrhage, hepatic coma, ascites, jaundice, and more than 2 of the above indicators. Alcohol dependence syndrome is related to chronic alcohol consumption, which has been previously determined to increase postoperative mortality [7, 34]. Liver function is usually assessed by the Child-Turcott-Pugh (CTP) classification and the Model for End-Stage Liver Disease (MELD) scores [13]. The Child-Turcott-Pugh classification considers five variables (serum bilirubin, albumin, prothrombin time, ascites, and hepatic encephalopathy), with higher classifications indicating worse prognosis in patients with cirrhosis [14]. Based on the above, our findings suggested that more severe ALD (with cirrhosis-related complications) increased 30-day in-hospital mortality in patients who underwent nonhepatic surgery.

The influence of ALD on perioperative outcomes is incompletely understood, and previous studies have focused on the effects of alcohol consumption instead of ALD [3, 7]. Although some studies mentioned that patients with liver cirrhosis have increased risks of postoperative complications and mortality [12, 35, 36], ALD does not consist of cirrhosis alone. ALD is the most prevalent type of chronic liver disease worldwide, and it can progress to alcoholic fatty liver and alcoholic steatohepatitis. Chronic alcoholic steatohepatitis eventually leads to fibrosis, cirrhosis, or hepatocellular carcinoma [24]. Our study described a more comprehensive design and explanation of the relationship between ALD and outcomes after nonhepatic surgery.

In conclusion, we found that patients with ALD had more complications and higher mortality after nonhepatic surgery compared with non-ALD controls, particularly those with severe liver symptoms. Further studies are needed to develop effective preventions for postoperative adverse events among patients with ALD.

## Supplementary Information

**Additional file 1. Table S1.** Codes of liver-related surgical procedure. **Table S2.** Liver-related characteristics of surgical patients with and without preoperative alcoholic liver disease. **Table S3.** Risks of postoperative mortality for surgical patients with the severity of alcoholic liver disease. **Table S4.** Risks of postoperative adverse events for surgical patients with the severity of alcoholic liver disease

## Data Availability

The data that support the findings of this study are available from the Ministry of Health and Welfare, Taiwan but restrictions apply to the availability of these data, which were used under license for the current study, and so are not publicly available. Data are however available from the authors upon reasonable request and with permission of the Ministry of Health and Welfare, Taiwan. We have made the formal application (included application documents, study proposals, and ethics approval of the institutional review board) of the current insurance data. The authors of the present study had no special access privileges in accessing the data which other interested researchers would not have.

## Abbreviations

ALD: Alcoholic liver diseases
CI: Confidence interval
ICD-9-CM: International Code of Diseases, Ninth Edition, Clinical Modification
OR: Odds ratio
