# Supplementary material for: Adverse outcomes after non-hepatic surgeries in patients with alcoholic liver diseases: a propensity-score matched study
Source: BMC Gastroenterol. 2022 Nov 21;22:475. doi: 10.1186/s12876-022-02558-6 (PMC9677632; doi:10.1186/s12876-022-02558-6)
Supplement: Supplementary file 1 — Additional file 1. Table S1. Codes of liver-related surgical procedure. Table S2. Liver-related characteristics of surgical patients with and without preoperative alcoholic liver disease. Table S3. Risks of postoperative mortality for surgical patients with the severity of alcoholic liver disease. Table S4. Risks of postoperative adverse events for surgical patients with the severity of alcoholic liver disease [file 12876_2022_2558_MOESM1_ESM.doc]

| **Table S1. Codes of liver-related surgical procedure** | |
| --- | --- |
| Code | Procedure |
| 75001B | Wedge biopsy of liver, laparotomy |
| 75002B | Partial hepatectomy; Segemental hepatectomy |
| 75003B | Hepatectomy: one segement |
| 75004B | Hepatectomy: two segements |
| 75005B | Hepatectomy: three segements |
| 75006B | Drainage or marsupialization of cyst or abscess of liver |
| 75007B | Hepatorrhaphy, suture of liver wound < 5cm |
| 75008B | Hepatorrhaphy, with common duct or gallbladder drainage |
| 75009B | Hepatorrhaphy, suture of liver wound,complicated or >5cm |
| 75010B | Hepatic artery ligation for liver bleeding |
| 75011B | Hepato-Enterostomy (Longmire Op.) |
| 75012B | Portocavo shunt (H-graft) |
| 75014B | Warren's shunt |
| 75015B | Right lobectomy |
| 75016B | Left lobectomy |
| 75017B | Extended right lobectomy |
| 75018B | Extended left lobectomy |
| 75019B | Hepaticotomy or hepaticostomy, Removal of Calculus |
| 75020B | Liver(Hepatic) transplantation |
| 75021B | Cadaveric liver harvest(donor hepatectomy) |
| 75022B | Partial hepatectomy for livingrelated liver transplantation |
| 75023B | Laparoscopic fenestration for hepatic cyst |
|  | |

| **Table S2. Liver-related characteristics of surgical patients with and without preoperative alcoholic liver disease** | | | | | |
| --- | --- | --- | --- | --- | --- |
|  | Alcoholic liver disease | | | | *p*-value |
|  | No (N=26802) | | Yes (N=26802) | |  |
| Medical conditions | n | (%) | n | (%) |  |
| Alcoholic fatty liver | 0 | (0.0) | 5511 | (20.9) | <0.0001 |
| Acute alcoholic hepatitis | 0 | (0.0) | 4380 | (16.6) | <0.0001 |
| Alcoholic cirrhosis of liver | 0 | (0.0) | 10378 | (39.3) | <0.0001 |
| Alcoholic liver damage | 0 | (0.0) | 9466 | (35.8) | <0.0001 |
| Alcohol dependence syndrome | 458 | (1.7) | 3824 | (14.5) | <0.0001 |
| Chronic hepatitis | 3379 | (12.6) | 12509 | (47.3) | <0.0001 |
| Liver cirrhosis | 1064 | (4.0) | 12409 | (47.0) | <0.0001 |
| Liver cancer | 571 | (2.1) | 1504 | (5.7) | <0.0001 |
|  | | | | | |

| **Table S3. Risks of postoperative mortality for surgical patients with the severity of alcoholic liver disease** | | | | | |
| --- | --- | --- | --- | --- | --- |
|  |  | 30-day in-hospital mortality | | | |
|  | n | Deaths | Mortality, % | OR | (95% CI)^a^ |
| Non-ALD controls | 26802 | 204 | 0.8 | 1.00 | (reference) |
| Patients with |  |  |  |  |  |
| Alcoholic fatty liver | 5511 | 30 | 0.5 | 1.00 | (0.68-1.48) |
| Acute alcoholic hepatitis | 4380 | 62 | 1.4 | 1.99 | (1.49-2.67) |
| Alcoholic cirrhosis of liver | 10378 | 361 | 3.5 | 3.97 | (3.32-4.74) |
| Alcoholic liver damage | 9466 | 143 | 1.5 | 2.05 | (1.65-2.55) |
| Alcohol dependence syndrome | 3824 | 106 | 2.8 | 3.13 | (2.44-4.02) |
| Chronic hepatitis | 12509 | 249 | 2.0 | 2.70 | (2.24-3.26) |
| Liver cirrhosis | 12409 | 367 | 3.0 | 3.58 | (3.00-4.26) |
| Liver cancer | 1504 | 34 | 2.3 | 2.08 | (1.43-3.04) |
| CI, confidence interval; OR, odds ratio.  ^a^Adjusted for all covariates listed in Table 1. | | | | | |

| **Table S4. Risks of postoperative adverse events for surgical patients with the severity of alcoholic liver disease** | | | | | |
| --- | --- | --- | --- | --- | --- |
|  |  | Adverse events | | | |
|  | n | Events | Incidence, % | OR | (95% CI)^a^ |
| Non-ALD controls | 26802 | 2506 | 9.4 | 1.00 | (reference) |
| Patients with |  |  |  |  |  |
| Alcoholic fatty liver | 5511 | 388 | 7.0 | 0.98 | (0.87-1.10) |
| Acute alcoholic hepatitis | 4380 | 515 | 11.8 | 1.36 | (1.23-1.51) |
| Alcoholic cirrhosis of liver | 10378 | 2045 | 19.7 | 2.04 | (1.91-2.18) |
| Alcoholic liver damage | 9466 | 1275 | 13.5 | 1.58 | (1.47-1.70) |
| Alcohol dependence syndrome | 3824 | 640 | 16.7 | 1.70 | (1.53-1.88) |
| Chronic hepatitis | 12509 | 1717 | 13.7 | 1.58 | (1.48-1.70) |
| Liver cirrhosis | 12409 | 2168 | 17.5 | 1.89 | (1.78-2.02) |
| Liver cancer | 1504 | 216 | 14.4 | 1.06 | (0.91-1.24) |
| CI, confidence interval; OR, odds ratio.  ^a^Adjusted for all covariates listed in Table 1.  Adverse events included with 30-day in-hospital mortality, Acute renal failure, Postoperative bleeding, Stroke, Septicemia and Pneumonia. | | | | | |
